# Editorial: Antimicrobial surfaces and airborne pathogens: the new frontiers in hospital safety

**DOI:** 10.3389/fmicb.2025.1752935

**Published:** 2026-01-05

**Authors:** Smagul Karazhanov, Luminita Andronic, Mariana Carmen Chifiriuc, Alina Bereanu

**Affiliations:** 1 Department for Solar Energy Materials and Technologies, Institute for Energy Technology, Kjeller, Norway; 2 Thin Film Laboratories, Institute of Solid State Physics, University of Latvia, Riga, Latvia; 3 Product Design and Environment Faculty, Product Design, Mechatronics and Environment Department, Transilvania University of Brasov, Brasov, Romania; 4 Faculty of Interdisciplinary Studies, University of Bucharest, Bucharest, Romania; 5 The Biological Sciences Division, Romanian Academy, Bucharest, Romania; 6 Faculty of Medicine, Lucian Blaga University of Sibiu, Sibiu, Romania; 7 County Clinical Emergency Hospital, Sibiu, Romania

**Keywords:** hospital-acquired infections (HAIs), antimicrobial surfaces, airborne pathogens, infection control strategies, ISO standards for antimicrobial testing, respiratory particle emission and transmission risk

Hospital-acquired infections (HAIs) remain a major global health challenge, contributing to increased morbidity, mortality, and healthcare costs. The COVID-19 pandemic further highlighted the critical role of surface contamination and airborne transmission in pathogen spread. As hospitals strive to improve infection control, antimicrobial surfaces and technologies targeting airborne pathogens have emerged as promising strategies to enhance patient safety and reduce cross-contamination. Healthcare environments are shaped by two intertwined challenges: persistent contamination of inanimate surfaces and airborne transmission of respiratory pathogens. This Research Topic, “*Antimicrobial Surfaces and Airborne Pathogens: The New Frontiers in Hospital Safety”*, brings together cutting-edge research addressing these urgent needs through innovative materials, engineering solutions, and microbiological insights.

Eleven papers have been submitted to the Research Topic. Among them six high-quality papers were accepted for publication, each contributing unique perspectives and advancements:

One of the research directions within this Research Topic was the development of novel materials and modalities to combat microbial colonization on hospital surfaces. Poelzl et al. benchmarked multiple non-porous coatings against the emerging multidrug-resistant yeast *Candidozyma auri*s. They show that zinc-based formulations aided by copper or silver achieve ≥3-log reductions after 24 h, while an antimicrobial lacquer achieves rapid antifungal activity within 1 h. The authors also show that *Candida albicans* closely tracks *C. auris* performance and can serve as a practical surrogate for surface testing. This work underscores the need to standardize realistic contact times and humidity conditions when claiming performance.

On the epidemiological front, Horstink et al. reviewed 44 human studies examining host-related factors that affect viral transmission via respiratory particle emission. Their analysis indicates that the release of fine particles (< 5 μm) increases with age, physical activity, and active infection. The presence of detectable viruses in exhaled particles correlates most strongly with time since symptom onset and lower-respiratory symptoms. Although methodological approaches varied across studies, the observed trends highlight practical indicators for managing airborne transmission risk.

Turning from chemical to physical antimicrobial strategies, Schaal et al. evaluate an indirect, contact-free plasma–aerosol antimicrobial strategy. *In vitro*, cold atmospheric plasma aerosols (CAP-A) yielded significant microbial reductions across standard strains, while in an *in vivo* skin model with *Escherichia coli*, CAP-A showed antibacterial efficacy comparable to an alcohol reference. This study demonstrates that CAP-A is a tolerable, novel approach for antimicrobial treatment that warrants head-to-head trials under harmonized protocols.

Ly-Sauerbrey et al. compared single-species tests with a mixed reference community on glass coated with varying copper levels. While pure copper produced the strongest killing effect, reduced copper alloys blunted performance. Notably, survival within the community exceeded single-species survival, especially for *Staphylococcus capitis*. The study highlights the importance of testing in microbial communities to capture real-world resilience.

Fijan et al. critically appraise “probiotic” cleaning products in healthcare. Under EU rulings, formulations containing live bacteria are considered biocidal products and require authorization; strikingly, none of the products studied in European hospitals were authorized, and many lacked strain-level identification. The field needs properly characterized strains, defined modes of action, and trials conducted within the existing regulatory framework.

In the field of methods and standards, Maitz et al. performed a comparative analysis of ISO 22196:2011 (wet-film, 24 h) and the newer ISO 7581:2023 (“dry test”) for the evaluation of antimicrobial surfaces. While ISO 7581 better mimics real use, low application volumes can hurt reproducibility, and not all strains are suitable for the method. Therefore, thoughtful protocol adaptations (volume, humidity, strain set) are often required. Nevertheless, when used properly, both standards are valuable baselines for estimating efficacy.

Read together, these six papers advance hospital safety by: (i) detailing novel materials and modalities for combating microbial contamination, (ii) clarifying the advantages and limitations of current methods and standards for assessing antimicrobial efficacy, and (iii) identifying indicators of transmission risk and individuals with elevated infectiousness.

We thank the authors and reviewers for moving the science and the implementation details forward. The road to safer hospitals runs through careful mechanistic work, realistic testing, and regulatory clarity. We also extend our sincere gratitude to the Editor-in-Chief of *Frontiers in Microbiology* for supporting this Special Issue and acknowledge the efforts of those whose submissions were not accepted; their work reflects the growing momentum in this critical research area. Together, the articles in this Research Topic represent a significant advancement toward reducing healthcare-associated infections (HAIs) and improving patient safety worldwide.

